# Metal Induced Growth of Transition Metal Dichalcogenides at Controlled Locations

**DOI:** 10.1038/srep38394

**Published:** 2016-12-02

**Authors:** Zhendong Wang, Qi Huang, Peng Chen, Shouhui Guo, Xiaoqing Liu, Xuelei Liang, Li Wang

**Affiliations:** 1 Department of Physics, Nanchang University, Nanchang 330031, China; 2 Key Laboratory for the Physics and Chemistry of Nanodevices and Department of Electronics, Peking University, Bejing 100871, China; 3 Nanoscale Science and Technology Laboratory, Institute for Advanced Study, Nanchang University, Nanchang 330031, China

## Abstract

Metal induced nucleation is adopted to achieve the growth of transition metal dichalcogenides at controlled locations. Ordered arrays of MoS_2_ and WS_2_ have successfully been fabricated on SiO_2_ substrates by using the patterned Pt/Ti dots as the nucleation sites. Uniform MoS_2_ monolayers with the adjustable size up to 50 μm are grown surrounding these metal patterns and the mobility of such layer is about 0.86 cm^2^/V·s. The crystalline flakes of WS_2_ are also fabricated extending from the metal patterns and the electron mobility of these flakes is up to 11.36 cm^2^/V·s.

Two-dimensional (2D) materials have attracted considerable interest due to their unique electrical, optical, thermal and mechanical properties that do not exist in their bulk form^1^,^2^,^3^,^4^,^5^,^6^,^7^,^8^,^9^,^10^. Thus 2D materials are regarded as potential candidates for future logic devices^11^, integrated circuits^12^, and optoelectronics devices^13^. Transition metal dichalcogenides (TMDs) are important 2D materials, which have been researched extensively. Although single and few-layer TMD have been synthesized by many methods^14^,^15^,^16^,^17^,^18^,^19^, the grown TMD materials are small flakes instead of a whole continuous film throughout the substrate and the flakes with various shapes distributed randomly on the substrate^20^,^21^,^22^,^23^, which limits the large scale devices fabrication and hence their applications.

An alternative is to grow TMD at predesigned locations where the electronic devices with certain function will be defined, which provides a promising way to overcome small crystal size and random distribution for TMD used in large scale devices fabrication. To our knowledge, there are very limited reports on the growth of TMD at defined locations. Han *et al*.^24^ declared that the crystalline MoS_2_ monolayer can be grown at predefined locations by using lithographically patterned islands of MoO_3_ or ammonium heptamolybdate as seed materials. Su *et al*.^25^ reported that the layered semiconductor SnS_2_ arrays was grown on the patterned substrate by using thin-film pads of Pd/Cr, Cr, SiO_2_/Cr, and Ni as nucleation sites. Godin *et al*.^26^ also reported the growth of polycrystalline WS_2_ monolayers on patterned substrates by controlling surface energy via oxygen-plasma treatment. Lou *et al*.^27^ found that the MoS_2_ triangular crystals are commonly nucleated and formed on the step edges of SiO_2_. However, it is still a challenge to grow various category transition metal dichalcogenides at controlled locations by a generic growth protocol.

In this study, metal induced nucleation is proposed to be a simple way to grow transition metal dichalcogenides at controlled locations. Ordered arrays of MoS_2_ and WS_2_ were successfully grown on SiO_2_ substrates by using the patterned Pt/Ti dots as the nucleation sites. Uniform MoS_2_ monolayers are grown surrounding the metal patterns and the size of the MoS_2_ monolayer can be easily controlled by adjusting the size of the used metal pattern. Moreover, the mobility of such MoS_2_ layer is measured to be about 0.86 cm^2^/V·s. Under the same protocol, the crystalline WS_2_ flakes are also grown extending from the metal patterns and the electron mobility of the flakes is up to 11.36 cm^2^/V·s.

## Results and Discussion

### Growth protocol of the 2D TMDs

**Figure 1:**
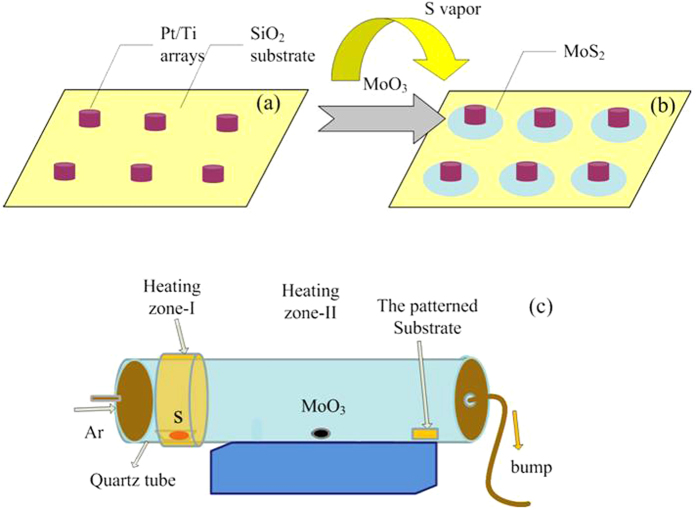
(**a,b**) Schematic of the growth process of the monolayer MoS_2_ arrays using the patterned Pt/Ti as cores deposited on SiO_2_/Si substrate, and (**c**) Experimental setup of the CVD system.

As shown in Fig. 1, the TMD are grown in a two-zone CVD furnace. The pre-patterned metal (Pt/Ti) arrays are fabricated on SiO_2_ covered Si wafer by e-beam lithography. Solid reactive precursors, sulfur powder and MoO_3_ powder (or the ball-milled WO_3_ and NaCl powder) were placed in the heating zone-I and zone-II, respectively. When the furnace was heated up, the solid precursors were sublimated into the quartz tube and transferred to the substrate by the carrier gas, Ar. The Pt/Ti patterns are expected to act as the nucleation sites for the growth of TMD.

### Characterization of MoS_2_

**Figure 2:**
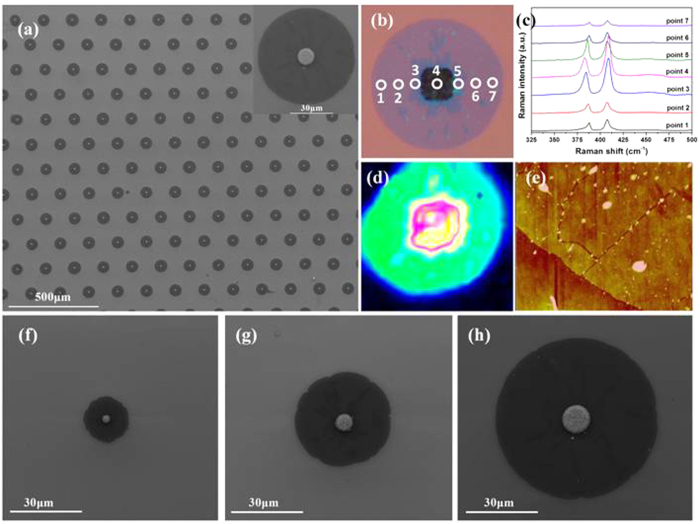
(**a**) SEM images of the patterned growth of the MoS_2_ layers. Inset: high magnification image of a typical MoS_2_ flake. (**b**) Optical image of a typical MoS_2_ flake (**c**) Raman spectra correspond to postions 1–7 in (**b**). (**d**) The *E*^*1*^_2g_ peak intensity mapping for the MoS_2_ flake in (**b**). Due to the sample drift during the Raman mapping measurement, the shape of the flake seems distorted a bit as compare to the optical image in (**b**). (**e**) AFM images of the edge of the MoS_2_ layer on the substrate, and (**f–h**) SEM images of the different dimensions MoS_2_ units.

Figure 2a shows a typical SEM image for MoS_2_ grown on the substrate by this metal induced nucleation method. It is obvious that the circular films exactly follow the patterned metal dots to form a well ordered array. The inset of Fig. 2a, the magnified image for a single unit, clearly demonstrates that the bright metal dot is in the middle of the dark circular film, indicating that this dark film actually grows from the metal dot. Figure 2c shows a series of Raman spectra taken at various points on a circular MoS_2_ film (shown in Fig. 2b). There are two prominent peaks at ~387 cm^−1^ and 407 cm^−1^ at all the Raman spectra. It is well known that these two peaks are ascribed to the in-plane mode *E*^1^_2g_ and out-of-plane mode *A*_1g_ of the MoS_2_, respectively. The presence of these two Raman peaks unambiguously gives the evidence that these dark films are indeed MoS_2_ grown on the substrate. On the other hand, these two Raman modes of *E*^*1*^_2g_ and *A*_1g_, are very sensitive to the layer thickness^28^,^29^, which provide a convenient and reliable means to determine the thickness of MoS_2_ film. The frequency difference value (Δ) between these two peaks are about 19.5 cm^−1^ ~20.0 cm^−1^ at the points of 1, 2, 6 and 7 in Fig. 2b, respectively, confirming that the thickness of the MoS_2_ film surrounding the Pt/Ti dots is one monolayer. The vibration modes of MoS_2_ are also observed in the spectrum taken at the metal dot (point 4 in Fig. 2b), indicating that the MoS_2_ is also grown on the top of the metal. Moreover, relative larger frequency differences (about 22∼27 cm^−1^) are obtained on the top and near the Pt/Ti dots, suggesting that the MoS_2_ films at these places are multilayers (Figure S2). The Raman mapping with the peak difference as the indicator (407 cm^−1^) given in Fig. 2d further reveal that the MoS_2_ film grown out of the Pt/Ti dot is very uniform monolayer. In addition, the PL spectrum in Figure S3 exhibits the strongest emission at ~1.83 eV for the MoS_2_ films grown around the metal dots, which is in agreement with the previous reports on MoS_2_ monolayer^30^,^31^. The thickness of the MoS_2_ film derived from AFM measurement in Fig. 2(e) is ~0.85 nm, which is also consistent with the values for MoS_2_ monolayer^32^, and the results also revealed that there are some cracks in the MoS_2_ units in Fig. 2(e), it is possible that the cracks are ascribe to the domain boundaries of the polycrystalline MoS_2_, where some domain boundaries of the polycrystalline MoS_2_ are slightly oxidized in air. It is worth noting that the dimension of MoS_2_ monolayer grown by this method can be easily controlled by adjusting the size of the metal dots. Figure 2f–h gives the SEM images of MoS_2_ monolayer grown around the metal dots with the various sizes under the same growth condition. A SiO_2_/Si substrate containing an array of the metal dots with various sizes was used to grow the MoS_2_ monolayer around various metal dots at the same time, as shown in Fig. 2f–h. It is obvious that the dimension of the MoS_2_ monolayer increases from 18.3 μm to 53.5 μm as the size of the metal dot varies from 3 μm to 10 μm. Careful examinations reveal that the area of the grown MoS_2_ film linearly depends on the circumference of the metal dot, indicating that the radial growth rate of MoS_2_ around the metal dot might be kept the same during the growth process. (See Figure S4).

**Figure 3:**
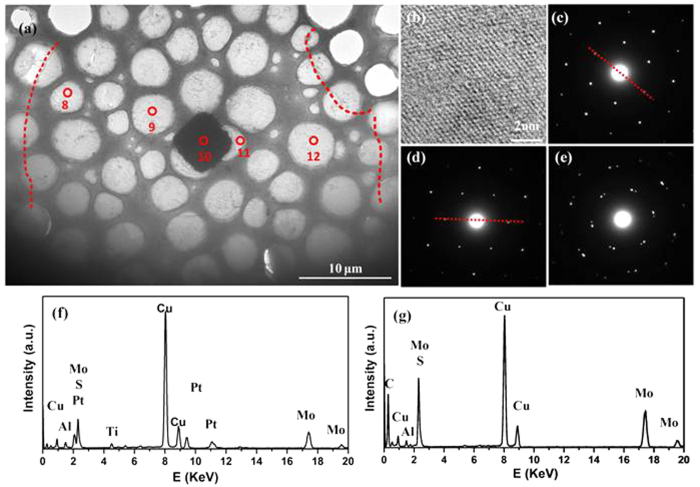
TEM images of the MoS_2_ units, TEM images of (**a**) the MoS_2_ unit and (**b**) the typical HRTEM image for the MoS_2_ unit, the SAED pattern for (**c**) and (**d**) the position of point 8 and point 9, respectively, (**e**) the position of point 10 and point 11, and EDS analysis for the position of (**f**) point 10, (**g**) point 11.

Transmission electron microscopy (TEM) was used to characterize the crystal structures of the obtained MoS_2_ film. Figure 3c shows the low magnification image of MoS_2_ film as well as the metal dot (the center black area) that transferred onto Cu grid. The boundary of the transferred the film was outlined by the red dashed line in Fig. 3a. Figure 3(b) shows a typical high-resolution TEM (HRTEM) image. The periodic atom arrangement is clearly observed and the specific inter-planar distances for the (100) plane is measured to be about 0.285 nm, which gives the direct evidence for the crystalline nature of such MoS_2_ monolayer. The selected area electron diffraction (SAED) was taken on different location as marked by the numbers in Fig. 3a. In Figure 3c and d, there is only one set of the hexagon diffraction pattern at location 8 and 12 but with different orientation, indicating that the MoS_2_ film in these areas are crystalline monolayer but in different crystalline domains. The cracks on the AFM image in Fig. 2e represent the domain boundaries between different domains, indicating the polycrystalline nature of the MoS_2_ film. The presence of two set of the hexagon spots in the SAED patterns for the areas of the point 10 and point11 (Fig. 3(e)) shows that the few-layers MoS_2_ is grown on the top and near the Pt/Ti dots. The element distribution in the MoS_2_ film is measured by energy dispersive spectroscopy (EDS). Although the elements Pt and Ti can be found in the multilayer area on the top of the metal dot (Fig. 3(f)), they are not observed in the regions far away from the metal dot (Fig. 3(g)), and only the S and Mo elements are measured. These results suggested that the metal dot only acts as a nucleation site and the metals, Pt and Ti, do not diffuse into the MoS_2_ film during the growth process, at least in our measurement accuracy.

### Characterization of WS_2_

**Figure 4:**
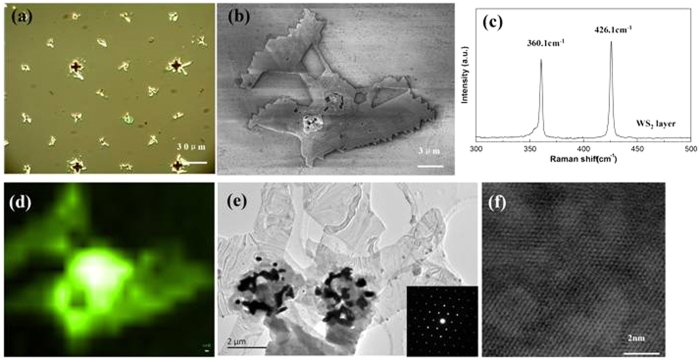
(**a**) Optical image of the WS_2_ layers induced grown on the substrate, (**b**) SEM image of the WS_2_ unit, (**c**) the typical Raman spectra of the WS_2_ unit, (**d**) the peak position maps for the WS_2_ unit, (**e**) TEM images of the WS_2_ layers and the inset for the typical images of the SAED pattern, (**f**) the typical HRTEM image of the WS_2_ layers.

WS_2_ films are also successfully grown on the substrate by using the same growth protocol. Figure 4(a) shows an optical image of the WS_2_ films grown on the prepatterned substrates in which the films appear much brighter than the metal dots. Although the WS_2_ films are not uniformly circular shape like the MoS_2_ films, these WS_2_ films certainly follow the periodicity of the metal pattern, indicating that the metal dots do act as the nucleation sites during the film growth process. The SEM image of the WS_2_ unit in Fig. 4(b) clearly shows that the irregular WS_2_ flakes extend from the center metal dot, in contrast to a symmetric circular shape of the MoS_2_ monolayer in Fig. 2a. The chemical reaction process different to those of MoS_2_ may account for the irregular shape of the WS_2_ grown around the metal dot. The typical Raman spectrum for the films is shown in Fig. 4(c). Two peaks located at 360.1 cm^−1^ and 426.1 cm^−1^ are observed, which are the well known *E*^*1*^_2g_ and *A*_1g_ modes for WS_2_^33^. Figure 4(d) shows the Raman mapping image for the WS_2_ unit in Fig. 4(b) by using the peak position (426.1 cm^−1^) as an indicator. The perfect match between the Raman mapping image and the SEM image directly reveals that the WS_2_ films are not grown on the bare substrate but around the metal dots, further confirming that the metal dots are indeed the nucleation sites for the growth of WS_2_ films. The sharp bright spots with a hexagonal periodicity in the SAED measurement for the WS_2_ flakes and the periodic atom arrangement with the specific interplanar distances of ~0.270 nm assigned to the (100) plane in the HRTEM image confirm the crystallinity nature of the WS_2_ flakes with high quality, as shown in Fig. 4(e) and (f).

### Electrical measurements

**Figure 5:**
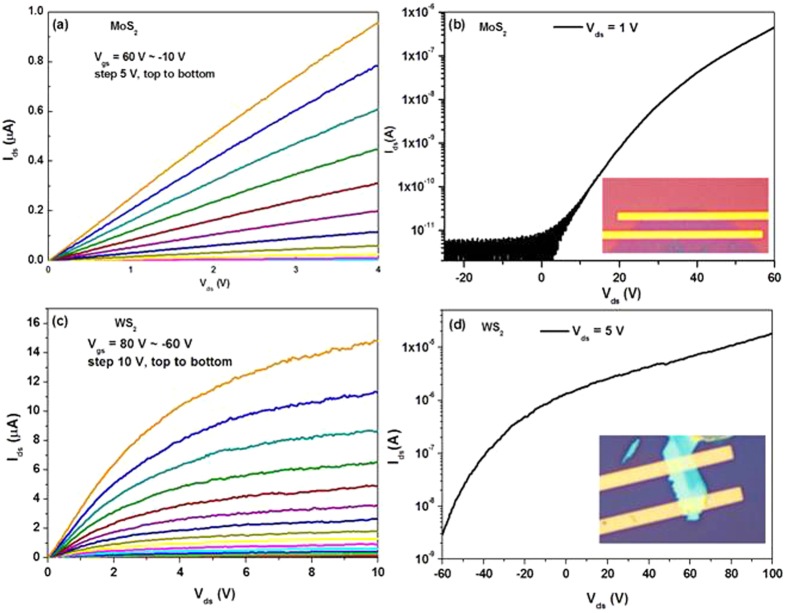
The electrical property of the measured MoS_2_ FETs (**a,b**) and WS_2_ FETs (**c,d**) The channel length and width are 2.82 μm and 24.5 μm for the MoS_2_ FETs, and 4.4 μm width and 4 μm length for the WS_2_ FETs.

In order to characterize the electronic properties of these grown TMD films, field effect transistors (FETs) were fabricated by e-beam lithography directly on the growth substrate without any transfer processes. Typical measurement results were shown in Fig. 5, where both the MoS_2_ and WS_2_ devices show good n-type field effects. The on/off ratio of the MoS_2_ FET is about 10^5^, while it is only about 10^4^ for the WS_2_ FET. The extracted electron mobility are 0.86 cm^2^/V·s for MoS_2_ and 11.36 cm^2^/V·s for WS_2_, respectively. The mobility of MoS_2_ is relatively lower as compared with the previous work^24^, which is originated from the polycrystallinite nature of the grown MoS_2_ monolayer. During the fabrication process of these devices, the FETs were purposively built far from the pre-defined Pt/Ti dots to avoid the influence of the metal dots. As a consequence, the channel of the MoS_2_ FET is actually a MoS_2_ monolayer and that of the WS_2_ FET is a multilayered WS_2_. Therefore, the above measured results are understandable because that the multilayered channel is more difficult to be switched off than a monolayered channel but the higher mobility can be achieved in multilayered channel. The electrical properties of the FETs based on the grown MoS_2_ and WS_2_ films unambiguously confirm the high quality of the TMD films grown by this metal indunced nucleation method. Such observations also support our argument that large scale devices fabrication can be easily achieved via the defined location growth of TMD.

In summary, we reported a metal induced growth method for the transition metal dichalcogenides grown at controlled locations. Where the high quality monolayer MoS_2_ arrays are grown orderly around the core of Pt/Ti patterned on the substrates, and the WS_2_ multilayers are also prepared at a controlled location by a simple CVD technology. The mobility of the MoS_2_ films and the crystalline WS_2_ flakes are about 0.86 cm^2^/V·s and up to 11.36 cm^2^/V·s, respectively. The results are hopeful for facilitating device fabrication for the integrated devices based on the transition metal dichalcogenides.

## Methods

### Growth process of the MoS_2_

Pure MoO_3_ powder was placed in a quartz boat at the centre of furnace, cleaned substrates with patterned Pt/Ti cores were placed on the downstream, and a separate quartz boat with sulfur powder was placed on the upstream, which was heated up to 190 °C using a separate heating system. The furnace was heated from room temperature to 850 °C at a ramp rate of 15 °C/min under an argon (99.999%) flow of 100 sccm. The temperature was held constant for 30 min during the MoS_2_ growth, and the furnace chamber was then rapidly cooled to room temperature by opening the furnace door.

### Growth process of the WS_2_

Pure WO_3_ powder and NaCl powder with the molar ratio of 1.4:1 were mixed and ball-milled in a grinding container for 2 h, using alcohol as a solvent, then dried at 95 °C^34^. According to the ref. 34, WO_3_ is the tungsten precursor but NaCl acts as a growth promoter. Afterwards the ball-milled powders were placed in a quartz boat at the centre of furnace, a separate quartz boat with sulfur powder was placed on the upstream, which was heated up to 190 °C using a separate heating system, and cleaned substrates with patterned Pt/Ti cores were placed on the downstream. The furnace was heated from room temperature to 900 °C at a ramp rate of 15 °C/min. The temperature was held constant for 30 min during the WS_2_ growth, and the furnace chamber was then rapidly cooled to room temperature by opening the furnace door.

### Device fabrication and testing

The MoS_2_ and WS_2_ field effect transistors were fabricated by e-beam lithography using Raith 150. After exposure, the source and drain electrodes (Ti/Au film of 10/30 nm thick) was deposited by using e-beam evaporator (K. J. Lesker with base vacuum of 7×10^−8^ torr) followed by lift-off process. The bottom Si was used as gate electrode. The filed effect properties of the fabricated devices were measured by using probe station and Keithley 4200 Semiconductor parameter analyzer at room temperature in air.

### Characterizations

The morphologies and microstructures of transition metal dichalcogenides were characterized by optical microscopy, scanning electron microscopy (FEI-Quanta 200 F), atomic force microscope (Veeco Dimension 3100) and transmission electron microscopy (JEOL JEM-2100 at 80 keV). Raman and PL spectra were taken by Horiba Jobin Yvon LabRAM H8000 system with laser excitation wavelength of 488 nm.

## Additional Information

**How to cite this article**: Wang, Z. *et al*. Metal Induced Growth of Transition Metal Dichalcogenides at Controlled Locations. *Sci. Rep.* **6**, 38394; doi: 10.1038/srep38394 (2016).

**Publisher's note:** Springer Nature remains neutral with regard to jurisdictional claims in published maps and institutional affiliations.

## Supplementary Material

Supplementary Information

## References

[b1] Mak K. F. . Atomically Thin MoS_2_: a New Direct-Gap Semiconductor. Phys. Rev. Lett. 105, 136805 (2010). 21230799 10.1103/PhysRevLett.105.136805

[b2] Zhang C. D. . Visualizing Band Offsets and Edge States in Bilayer–Monolayer Transition Metal Dichalcogenides Lateral Heterojunction. Nat. Commun. 7, 10349 (2016). 10.1038/ncomms10349 PMC4735610 26778119

[b3] Wang Q. H. . Electronics and Optoelectronics of Two-Dimensional Transition Metal Dichalcogenides. Nat. Nanotechnol. 7, 699–712 (2012). 23132225 10.1038/nnano.2012.193

[b4] Yoo Y. d., Degregorio Zachary P. & Johns James E. Seed Crystal Homogeneity Controls Lateral and Vertical Heteroepitaxy of Monolayer MoS_2_ and WS_2_. J. Am. Chem. Soc. 137, 14281–14287 (2015). 26488069 10.1021/jacs.5b06643

[b5] Wu W. Z. . Piezoelectricity of Single-Atomic-Layer MoS_2_ for Energy Conversion and Piezotronics. Nature 514, 470–474 (2014). 25317560 10.1038/nature13792

[b6] Geim A. K. & Grigorieva I. V. Van der Waals Heterostructures. Nature 499, 419–425 (2013). 23887427 10.1038/nature12385

[b7] Lv R. . Two-Dimensional Transition Metal Dichalcogenides: Clusters, Ribbons, Sheets and More. Nano Today 10, 559–592 (2015).

[b8] Chen X. . Growth of Triangle-Shape Graphene on Cu(111) Surface. App. Phys. Lett. 100, 163106 (2012).

[b9] Wang C. C. . Growth of Millimeter-Size Single Crystal Graphene on Cu Foils by Circumfluence Chemical Vapor Deposition. Sci. Rep. 4, 4537 (2014). 24686949 10.1038/srep04537 PMC3971397

[b10] Najmaei S. . Vapour Phase Growth and Grain Boundary Structure of Molybdenum Disulphide Atomic Layers. Nat. Mater. 12, 754–759 (2013). 23749265 10.1038/nmat3673

[b11] Gong C. . Band Alignment of Two-Dimensional Transition Metal Dichalcogenides: Application in Tunnel Field Effect Transistors. Appl. Phys. Lett. 103, 053513 (2013).

[b12] Radisavljevic B., Whitwick M. B. & Kis A. Integrated Circuits and Logic Operations Based on Single-Layer MoS_2_. ACS Nano 5, 9934–9938 (2011). 22073905 10.1021/nn203715c

[b13] Yin Z. . Single-Layer MoS_2_ Phototransistors. ACS Nano 6, 74–80 (2012). 22165908 10.1021/nn2024557

[b14] Gao Y. . Large-Area Synthesis of High-Quality and Uniform Monolayer WS_2_ on Reusable Au Foils. Nat. commun. 6, 8569 (2015). 26450174 10.1038/ncomms9569 PMC4633959

[b15] Kim Y. J. . Self-Limiting Layer Synthesis of Transition Metal Dichalcogenides. Sci. Rep. 6, 18754 (2016). 26725854 10.1038/srep18754 PMC4698672

[b16] Wu S. F. . Vapor-Solid Growth of High Optical Quality MoS_2_ Monolayers with Near-Unity Valley Polarization. ACS Nano 7(3), 2768–2772 (2013). 23427810 10.1021/nn4002038

[b17] Kong D. . Synthesis of MoS_2_ and MoSe_2_ Films with Vertically Aligned Layers. Nano Lett. 13, 1341–1347 (2013). 23387444 10.1021/nl400258t

[b18] Liu K. K. . Growth of Large-Area and Highly Crystalline MoS_2_ Thin Layers on Insulating Substrates. Nano Lett. 12, 1538–1544 (2012). 22369470 10.1021/nl2043612

[b19] Su S. H. . Two-Dimensional Transition Metal Dichalcogenides via Vapour Deposition Techniques. Small 10, 2589–2594 (2014).24610642

[b20] Chen L. . Step-Edge-Guided Nucleation and Growth of Aligned WSe_2_ on Sapphire via a Layer-over-Layer Growth Mode. ACS Nano 9(8), 8368–8375 (2015). 26221865 10.1021/acsnano.5b03043

[b21] Ling X. . Role of the Seeding Promoter in MoS_2_ Growth by Chemical Vapor Deposition. Nano Lett. 14, 464−472 (2014). 24475747 10.1021/nl4033704

[b22] Mann J. . 2-Dimensional Transition Metal Dichalcogenides with Tunable Direct Band Gaps: MoS_2(1−x)_ Se_2x_ Monolayers. Adv. Mater. 26, 1399–1404 (2014). 24339159 10.1002/adma.201304389

[b23] Dumcenco D. . Large-Area Epitaxial Monolayer MoS_2_. ACS Nano 9(4), 4611–4620 (2015). 25843548 10.1021/acsnano.5b01281 PMC4415455

[b24] Han G. H. . Seeded Growth of Highly Crystalline Molybdenum Disulphide Monolayers at Controlled Locations. Nat. commun. 6, 6128 (2014). 10.1038/ncomms7128 25630052

[b25] Su G. X. . Chemical Vapor Deposition of Thin Crystals of Layered Semiconductor SnS_2_ for Fast Photodetection Application. Nano Lett. 15, 506–513 (2015). 25494406 10.1021/nl503857r

[b26] Godin K. . Increased monolayer domain size and patterned growth of tungsten disulfide through controlling surface energy of substrates. J. Phys. D: Appl. Phys. 49, 325304 (2016).

[b27] Najmaei S. . Vapour Phase Growth and Grain Boundary Structure of Molybdenum Disulphide Atomic Layers. Nat. Mater. 12, 754–759 (2013). 23749265 10.1038/nmat3673

[b28] Lee C. G. . Anomalous Lattice Vibrations of Single and Few-Layer MoS_2_. ACS Nano 4(5), 2695–2700 (2010). 20392077 10.1021/nn1003937

[b29] Li H. . From Bulk to Monolayer MoS_2_: Evolution of Raman Scattering. Adv. Funct. Mater. 22, 1385–1390 (2012).

[b30] Ganatra R. & Zhang Q. Few-Layer MoS_2_: A Promising Layered Semiconductor. ACS Nano. 8(5), 4074–4099 (2014). 24660756 10.1021/nn405938z

[b31] Ataca C., Topsakal M., Aktürk E. & Ciraci S. a Comparative Study of Lattice Dynamics of Three and Two Dimensional MoS_2_. J. Phys. Chem. C 115(33), 16354–16361 (2011).

[b32] Zhang J. . Scalable Growth of High-Quality Polycrystalline MoS_2_ Monolayers on SiO_2_ with Tunable Grain Sizes. ACS Nano 8(6), 6024–6030 (2014). 24818518 10.1021/nn5020819

[b33] Molina-Sánchez A. & Wirtz L. Phonons in Single-Layer and Few-Layer MoS_2_ and WS_2_. Phys. Rev. B 84, 155413 (2011).

[b34] Li S. S. . Halide-Assisted Atmospheric Pressure Growth of Large WSe_2_ and WS_2_ Monolayer Crystals. Appl. Mater. Today 1, 6066 (2015).

