# Supplementary material for: Metal Induced Growth of Transition Metal Dichalcogenides at Controlled Locations
Source: Sci Rep. 2016 Dec 2;6:38394. doi: 10.1038/srep38394 (PMC5133539; doi:10.1038/srep38394)
Supplement: Supplementary Information [file srep38394-s1.doc]

**Supplementary information**

**Metal Induced Growth of** **Transition Metal Dichalcogenides at Controlled Locations**

Zhendong Wang^1,§^, Qi Huang^2, §^, Peng Chen^1,§^, Shouhui Guo^1^, Xiaoqing Liu^1^, Xuelei Liang^2,*^, Li Wang^1,3,*^

^1^*Department of Physics, Nanchang University, Nanchang 330031, China*

^2^*Key Laboratory for the Physics and Chemistry of Nanodevices and Department of Electronics, Peking University, Bejing 100871, China*

^3^*Nanoscale Science and Technology Laboratory, Institute for Advanced Study, Nanchang University, Nanchang 330031, China*

^§^*These* *authors contributed equally*

^*^*Corresponding authors:* [*liwang@ncu.edu.cn*](mailto:liwang@ncu.edu.cn) *(Li Wang);* [*liangxl@pku.edu.cn*](mailto:liangxl@pku.edu.cn) *(Xuelei Liang)*


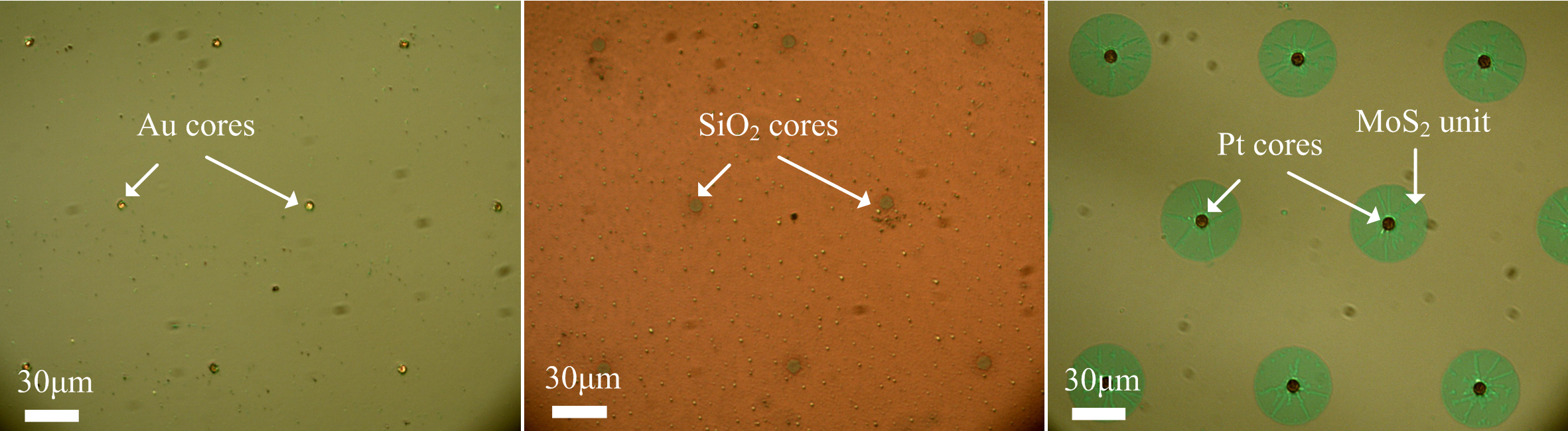


Figure S1｜Optical images of the induced growth of the MoS_2_ layer using different materials as the cores, (a) Au/Ti, (b) SiO_2_, and (c) Pt/Ti.

There is no MoS_2_ layer grown near Au and SiO_2_ cores in Figure S1 (a) and Figure S1 (b). However it is clearly shown that the circular MoS_2_ layer is grown at the controlled Pt locations. In other words, the MoS_2_ layer is only induced to grow by Pt metal. The Pt patterns might firstly react with S atom at high temperature and low pressure and then capture Mo atom to form MoS_2_. Au and SiO_2_ can’t act as Pt due to different chemical properties.


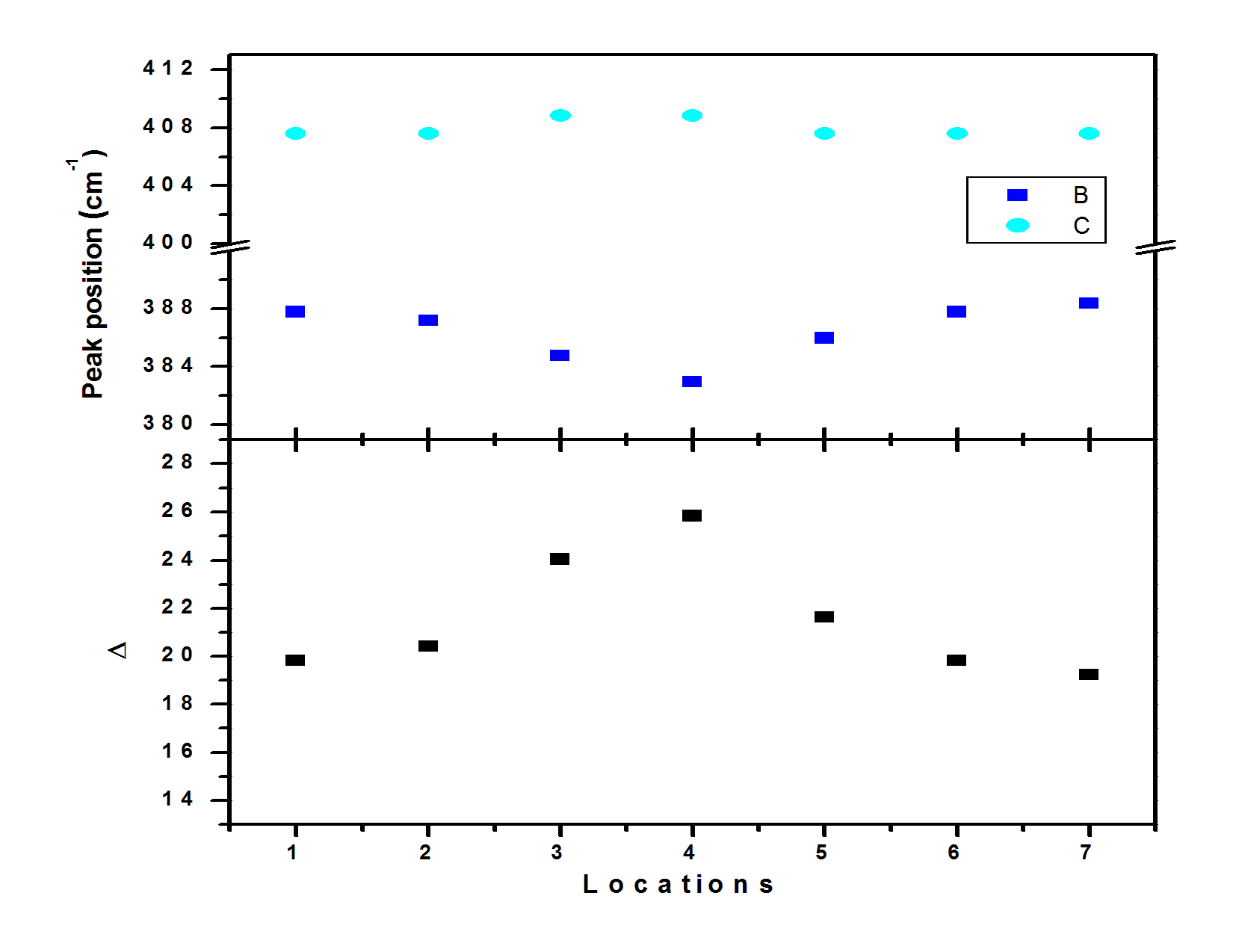


Figure S2 ｜The peak position and frequency difference (Δ) value of the Raman peaks for the MoS_2_ layer are obtained from different locations.


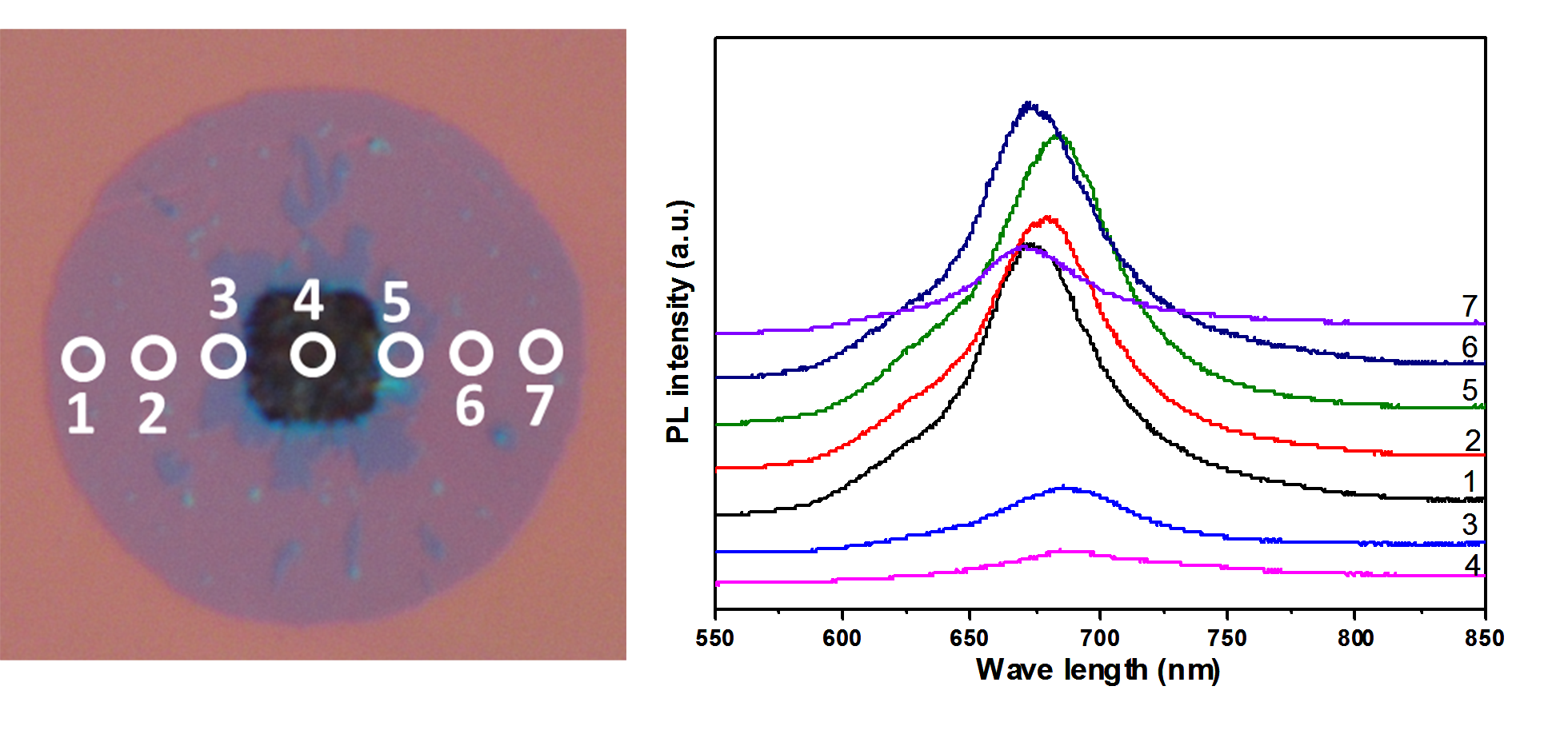


Figure S3｜(a) Optical image of the MoS_2_ unit, and (b) the PL spectra of the MoS_2_ unit performed on different point.

**
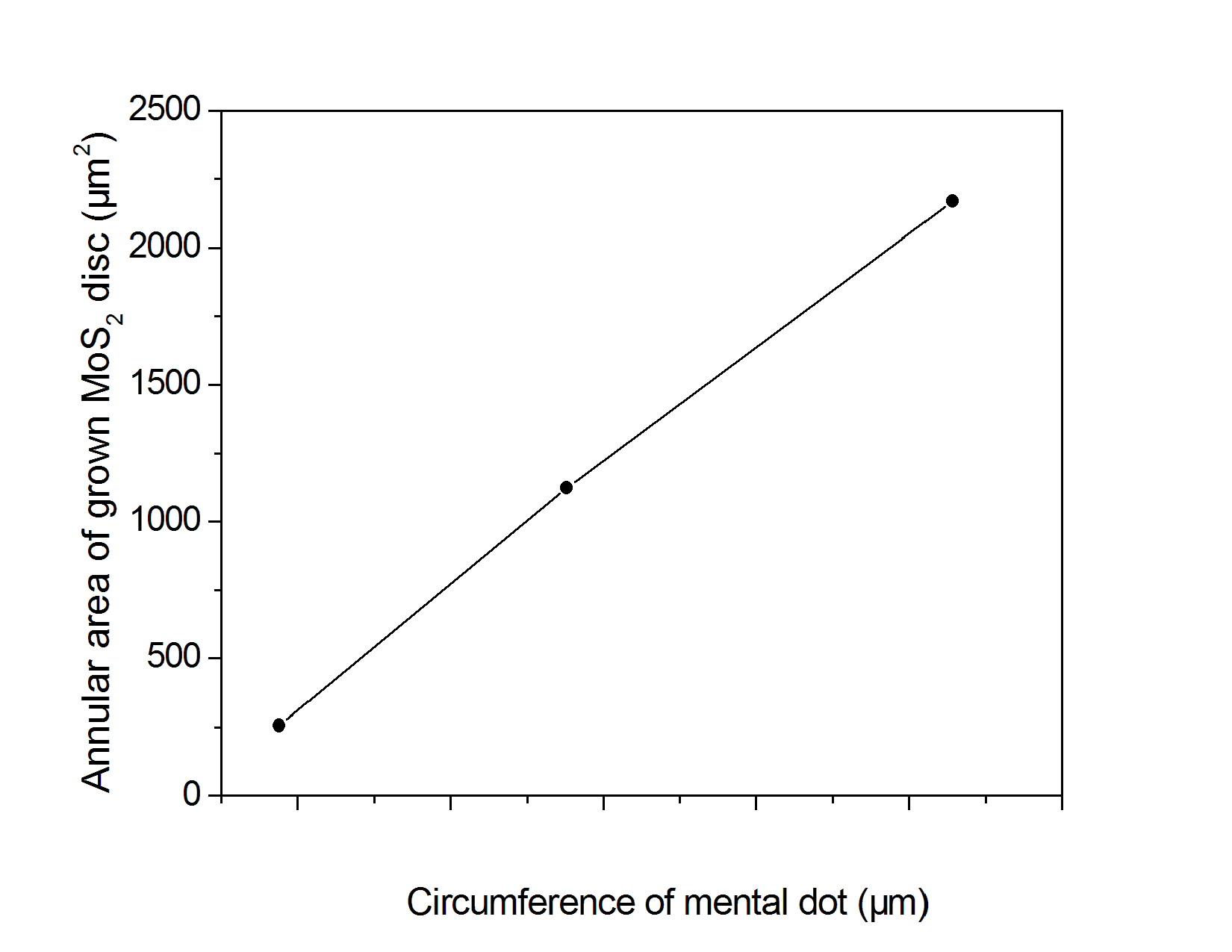
**

Figure S4｜The relationship between annular area of the grown MoS_2_ disc and the circumference of mental dot.


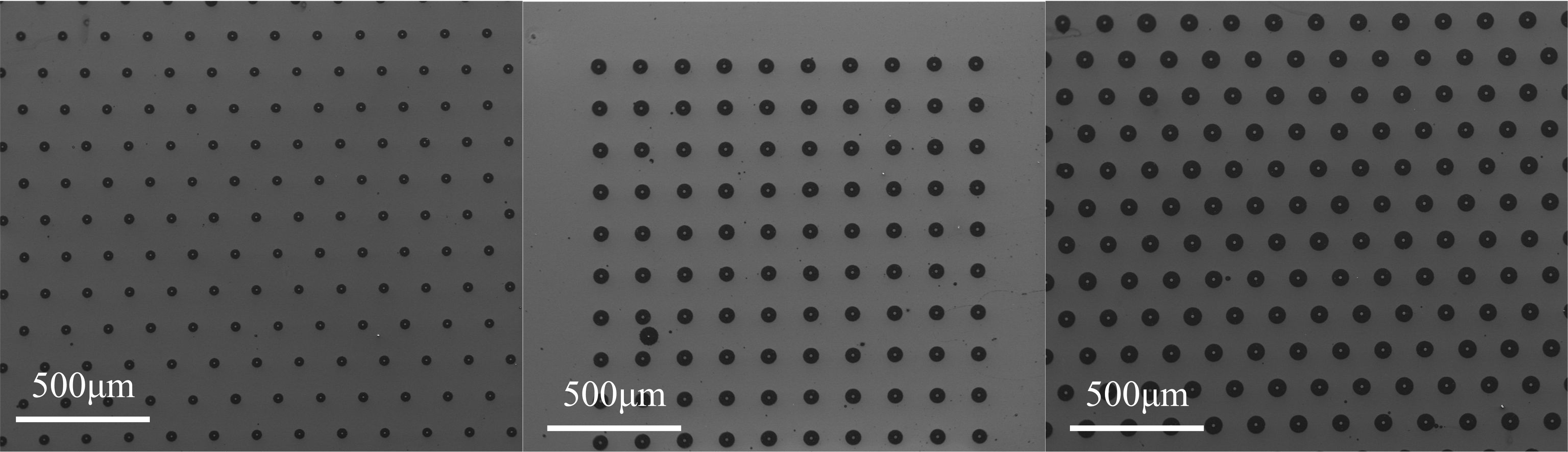


Figure S5｜SEM images of large area uniform MoS_2_ arrays with the controllable dimension. Where the MoS_2_ arrays was prepared with the same parameters: furnace temperature of 850°C, Ar : H_2_ = 100:3 (in the unit of sccm) and the growth time of 30 min, and the dimension is tunable by adjusting the size of the metal dots, (a) 3μm; (b) 6μm and (c) 10μm.


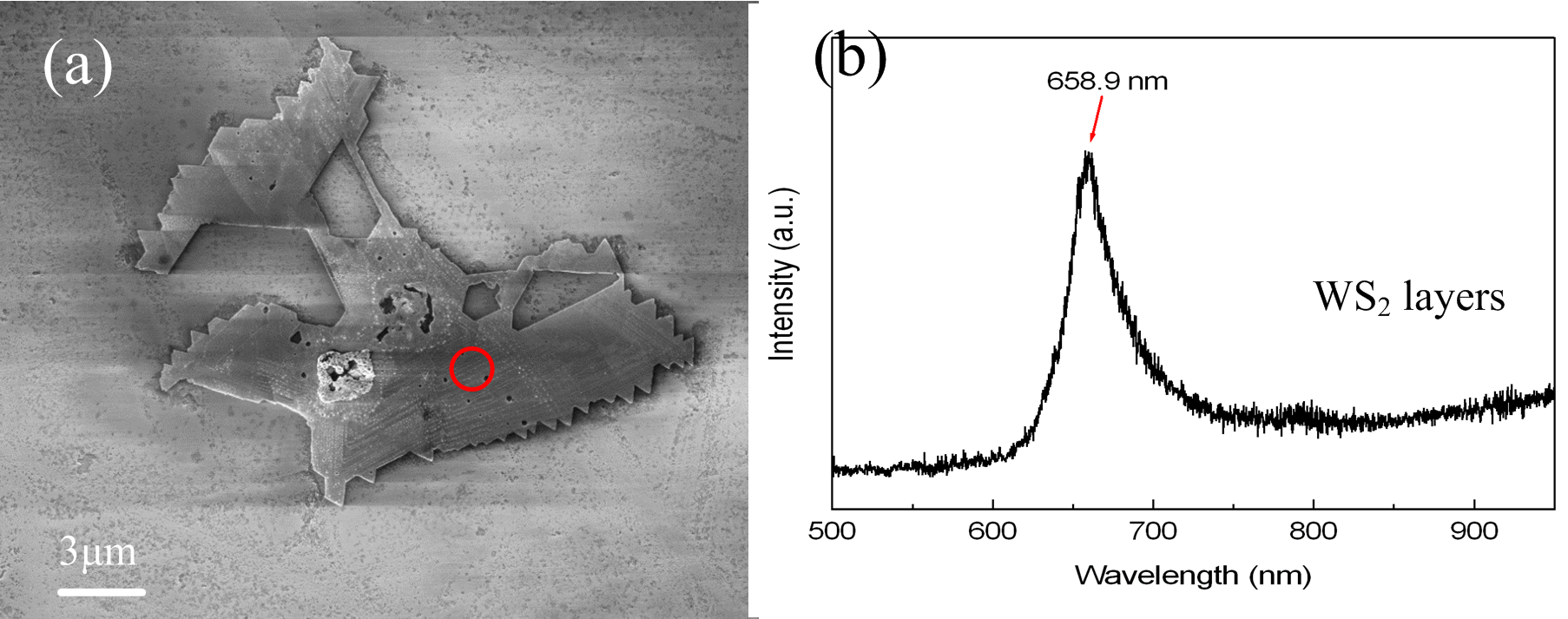


Figure S6｜(a) SEM image of the WS_2_ unit grown at controlled locations, and (b) the typical PL spectrum performed on the area of red circle.

Line scan profile across the edge of the MoS_2_ monolayer and WS_2_ are shown in Figure S7. The thickness of the MoS_2_ and WS_2_ film derived from AFM measurement is ～ 0.9 nm and 12.0 nm, respectively. It can be concluded that the MoS_2_ film is monolayer, and the number of WS_2_ multilayer is about 19 layers. The particles observed on the surface of MoS_2_ are scattered in multilayer MoS_2_ areas.


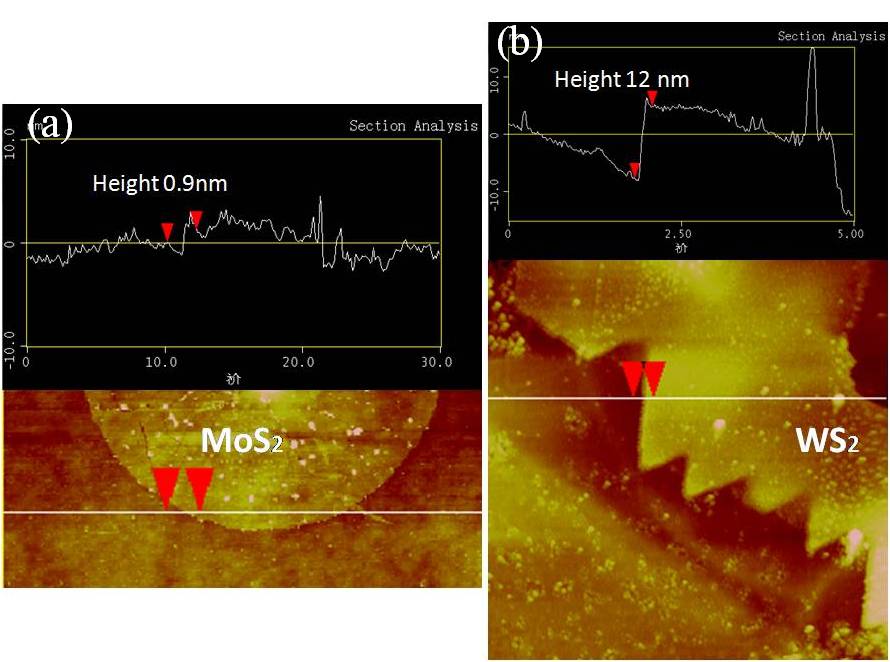


Figure S7｜AFM images of Line scan profile across the edge of (a) the MoS_2_ monolayer and (b) the WS_2_ multiplayer.

**The method of the sample transferred to Cu grid:** Firstly, a drop of isopropanol (IPA) is gently place on the top of the SiO_2_ /Si substrate and the Cu grid is put on the top of the substrate. Secondly, the Cu grid/IPA/substrate is baked at 100°C for 5 min for removing the IPA and making the MoS_2_ (WS_2_) and Cu grid bond together. Thirdly, the SiO_2_ layer is etched by floating the sample in a solution of 1% HF for 5 min. Finally, the Cu grid floated on DI water is take out and dried at 70 °C for 5 min.
